# Dietary Patterns in Relation to Components of Dyslipidemia and Fasting Plasma Glucose in Adults with Dyslipidemia and Elevated Fasting Plasma Glucose in Taiwan

**DOI:** 10.3390/nu11040845

**Published:** 2019-04-14

**Authors:** Li-Yin Lin, Chien-Yeh Hsu, Hsiu-An Lee, Wan-Hsiang Wang, Adi Lukas Kurniawan, Jane C.-J. Chao

**Affiliations:** 1 School of Nutrition and Health Sciences, College of Nutrition, Taipei Medical University, 250 Wu-Hsing Street, Taipei 11031, Taiwan; jlin11025@gmail.com (L.-Y.L.); adilukaskurniawan@yahoo.com (A.L.K.); 2 Department of Information Management, National Taipei University of Nursing and Health Sciences, 365 Ming-Te Road, Peitou District, Taipei 11031, Taiwan; cyhsu@ntunhs.edu.tw (C.-Y.H.); linda1598991@gmail.com (W.-H.W.); 3 Master Program in Global Health and Development, College of Public Health, Taipei Medical University, 250 Wu-Hsing Street, Taipei 11031, Taiwan; 4 Department of Computer Science and Information Engineering, Tamkang University, New Taipei 25137, Taiwan; billy72325@gmail.com; 5 Nutrition Research Center, Taipei Medical University Hospital, 252 Wu-Hsing Street, Taipei 11031, Taiwan

**Keywords:** dietary patterns, factor analysis, dyslipidemia, abnormal fasting plasma glucose, cross-sectional study, Taiwan

## Abstract

Dietary patterns have been proposed to be related to dyslipidemia and hyperglycemia. This study investigated the correlation of dietary patterns with components of dyslipidemia and fasting plasma glucose (FPG) among young and middle-aged adults (aged between 20 and 50 years) with dyslipidemia and abnormal FPG in Taiwan. This cross-sectional study used the database compiled in Taiwan between 2001 to 2010. A total of 13,609 subjects aged between 20 and 50 years were selected. Dyslipidemia was defined primarily according to the National Cholesterol Education Program Adult Treatment Panel III guidelines with minor modification. Elevated FPG level was defined according to the American Diabetes Association. The factor analysis was conducted to identify three dietary patterns. Higher scores of the meat–convenience dietary pattern (high intake of deep-fried and processed food, sauces, sugar-added beverages, meat and organ meats, instant noodles, rice or flour cooked in oil, and eggs) had no association with components of dyslipidemia and abnormal FPG. Higher scores of the vegetables–fruits–seafood dietary pattern (high intake of vegetables, vegetables with oil or dressing, fruits, seafood, legumes, soy products, and rice or flour products) was inversely associated with hypercholesterolemia and positively associated with hyperglycemia. Higher scores of the dairy–complex carbohydrate dietary pattern (high intake of dairy products, milk, root crops, jam or honey, and whole grains) was inversely correlated with hypertriglycemia and low high-density lipoprotein cholesterol level. Our results support that the dietary pattern may have a role in the prevention and management of dyslipidemia and abnormal fasting plasma glucose.

## 1. Introduction

Dyslipidemia and hyperglycemia are important risk factors for cardiovascular health. As these two diseases have significant genetic components, individuals with a family history of dyslipidemia or hyperglycemia are at higher risk for developing these two diseases [1,2]. Moreover, robust research has shown that unhealthy dietary habits, such as consuming excessive amounts of refined carbohydrates, sugar, and saturated and trans fats, increased the risk of developing dyslipidemia and hyperglycemia [3,4]. Dyslipidemia is a major predisposing factor for atherosclerotic cardiovascular disease in the general population and in hyperglycemic patients [5,6,7,8]. Dyslipidemia is defined as an abnormal lipid profile including elevated triglycerides (TG), total cholesterol (TC), low-density lipoprotein cholesterol (LDL-C), or low high-density lipoprotein cholesterol (HDL-C) [9,10,11]. According to the U.S. National Health and Nutrition Examination Survey, the prevalence of dyslipidemia was 53% in the U.S. adult population [12]. Mortality rate due to cardiovascular disease and stroke was up to four times higher in individuals with diabetes compared with those without diabetes [13].

Prevalence of dyslipidemia in Taiwan has been increasing mainly due to the changes of dietary habits and lifestyle, such as eating out more often and being less active. The Nutrition and Health Survey in Taiwan (NAHSIT) conducted between 2013 and 2015 found the prevalence of hyperlipidemia, hyperglycemia, and hypertension in adults (aged ≥20 years) was 23.7%, 12.3%, and 25.6%, respectively. The prevalence of these three conditions was increasing and the average age at diagnosis was getting lower, according to the epidemiological data reported by the Ministry of Health and Welfare in Taiwan [14]. Hyperlipidemia is considered a form of dyslipidemia that is characterized by abnormally elevated lipid levels [15]. The main contributors to this undesired health status are believed to be Westernized dietary habits, inactive lifestyle, and obesity [16]. In order to reduce the prevalence of hyperlipidemia, some lifestyle modifications and medical interventions are warranted. Reviews in the Cochrane Database found that dietary changes such as reducing the intake of saturated fat could effectively lower blood lipids and decrease incidence of cardiovascular disease and its associated mortality [17,18]. The NAHSIT surveys in 1993–1996 and 2005–2008 showed the Taiwanese dietary pattern was transforming into one that was high in refined bakery products and desserts, sugar-added beverages, and deep-fried or fried foods [19,20]. The sedentary lifestyle also became the norm for most people. The consequences of adapting such an unhealthy dietary pattern and lifestyle were consuming excessive calories and increasing the risk of developing obesity and other metabolic diseases such as metabolic syndrome, diabetes, hypertriglyceridemia, dyslipidemia, and gout [20,21].

Hyperglycemia is another worldwide public health concern worldwide. Impaired glucose tolerance (IGT) and impaired fasting glycemia (IFG) are often known as prediabetic conditions. The definition of prediabetes according to the American Diabetes Association in 2003 is as follows: IFG: 5.6–6.9 mmol/L, or IGT: impaired 2-hour post-glucose load of 7.8–11.1 mmol/L [22]. People with IGT or IFG were at high risk of developing type 2 diabetes and cardiovascular disease [22,23,24,25]. Lifestyle interventions are highly effective in delaying or preventing the onset of diabetes in people with IGT and may also reduce cardiovascular disease (CVD) and total mortality [23,26]. Many diabetes-related studies have found that early modifications in lifestyle, particularly diet and exercise, were effective in delaying the onset of diabetes [26].

Since diet is considered a complex exposure factor, multiple approaches are recommended in order to thoroughly examine the relationship between diet and disease risk. The traditional approach to investigate diet-and-disease associations usually focuses on single dietary components, for example, single nutrients or food items. However, in reality, individuals usually consume combinations of foods as meals rather than single food items, which makes it difficult to analyze the effects of dietary factors. Recent studies provided an estimation of food consumption of an individual’s dietary habits by using dietary pattern analysis. The two commonly used methods are factor analysis and cluster analysis, which are both data-driven methods, and these methods do not rely on the authors’ interpretation of a healthy dietary pattern [27]. Previous studies found a significant correlation of dietary patterns with CVD in Taiwanese middle-aged and/or older adults [28,29]. In prospective cohort studies, the Mediterranean diet was associated with lower risk for cancer, type 2 diabetes, and CVD [30,31]. Moreover, vegetarian and vegan diets were inversely related to abnormal fasting plasma glucose (FPG), while the Westernized diet was positively associated with elevated blood triglycerides [28,32,33,34]. There is no published study investigating the correlation of dietary patterns with dyslipidemia and abnormal fasting plasma glucose in Taiwan. The objective of this study was to investigate the correlation of dietary patterns with components of dyslipidemia and FPG among young and middle-aged adults (aged between 20 and 50 years) with dyslipidemia and abnormal FPG in Taiwan.

## 2. Materials and Methods

### 2.1. Study Design

The study was a cross-sectional approach using the data collected from the Mei Jau (MJ) International Health Management Institution in Taiwan between 2001 and 2010. The MJ International Health Management Institution is an independent health screening and management institution in Taiwan and consists of eight screening centers in Asia, four of which are located in major cities (Taipei, Tayouan, Taichung, and Kaohsiung) in Taiwan. Upon arriving at the health screening center, a structured questionnaire was given to the participants to collect information regarding demographics, medical history, diet, lifestyle, and exercise habits. A written consent form was given to the participants, which allowed the MJ International Health Management Institution to collect data for research purposes only, and personal information was kept confidential [28,29,35]. This study was approved by the Taiwan Medical University Joint Institutional Review Board (N201810008).

### 2.2. Participants and Data Collection

Initially, the MJ database included 765,064 adults who visited the MJ Health Management Institution for health screening between 2001 and 2010. We retrieved 96,088 subjects who were aged between 20 and 50 years and had both dyslipidemia and elevated fasting plasma glucose. Subjects who had family history of hyperlipidemia and diabetes; or with chronic disease such as cancer, liver disorder, renal disorder, or diabetes mellitus; or on thyroid, antivirus, steroid, diabetic, or cardiovascular-related medication (*n* = 66,620) were excluded. After removing those with missing data (*n* = 15,859), a total of 13,609 subjects were analyzed in this study.

### 2.3. Definition of Dyslipidemia and Elevated Fasting Plasma Glucose

According to the National Cholesterol Education Program Adult Treatment Panel III guideline and the “cutoff” value for abnormal lipid levels in Taiwan, dyslipidemia was originally defined as meeting one of the following: (1) elevated TG ≥ 2.3 mmol/L (200 mg/dL), (2) elevated TC ≥ 6.2 mmol/L (240 mg/dL), or (3) elevated LDL-C ≥ 4.1 mmol/L (160 mg/dL) [15]. In our study, we modified the definition of dyslipidemia as having “borderline” high lipid levels instead: (1) elevated TG ≥ 1.7 mmol/L (150 mg/dL), (2) elevated TC ≥ 5.2 mmol/L (200 mg/dL), or (3) elevated LDL-C ≥ 3.4 mmol/L (130 mg/dL). Furthermore, FPG ≥ 5.6 mmol/L (100 mg/dL) was defined as elevated blood glucose according to the American Diabetes Association [36].

### 2.4. Sociodemographic, Lifestyle, and Anthropometric Measurements

Sociodemographic data, including gender, age, education (below university level and university level or above), and marital status (single, married, and divorced or widowed), were obtained by self-reported questionnaire. Subjects reporting smoking cigarettes on a daily basis, sometimes, or occasionally during the study period were classified as current smokers (yes), while the rest were considered as nonsmokers (no). Drinking status was classified as drinking alcohol <2 times/week or ≥2 times/week. Physical activity was classified as <150 min/week or ≥150 min/week [28]. Anthropometric parameters such as height, weight, and body mass index (BMI) were measured during the health check-up as mentioned in the previous study [35].

### 2.5. Biochemical Profile

All serum blood samples were analyzed at the central laboratory of the MJ International Health Management Institution. Subjects were asked to fast overnight for 12–14 hours prior to blood being drawn. Concentrations of TG, TC, HDL-C, and FPG were measured using the commercial reagents [28], and LDL-C concentrations were calculated using the Friedewald formula [37].

### 2.6. Dietary Assessment

Food consumption data were collected by self-reporting using a standardized and validated semiquantitative food frequency questionnaire (FFQ) [28,29,35]. The FFQ contained 22 food items featuring typical Taiwanese dietary patterns. The participants were asked to answer how frequently they consumed certain portions of each food item in the past month (i.e., servings per day or per week from the lowest to the highest frequency) [28]. For example, the questions for consumption of light- or dark-colored vegetables, vegetables with oil or dressing, or root crops had 5 response options: <0.5 bowls/day, 0.5–1 bowls/day, 1–1.5 bowls/day, 1.5–2 bowls/day, and 2 bowls/day (1 bowl = 11 cm in diameter). For milk consumption, the 5 response options were: none or less than 1 glass/week, 1–3 glasses/week, 4–6 glasses/week, 1 glass/day, and 2 or more glasses/day (1 glass = 240 mL). For consumption of fruits and rice/flour products, the 5 response options were: <1 serving/day, 1–2 servings/day, 2–3 servings/day, 3–4 servings/day, and ≥4 servings/day (1 serving = 1 medium-sized apple, 1 bowl of rice, 2 bowls of noodles). For intake of other food items, the 5 response options were: <1 serving/week, 1–3 servings/week, 4–6 servings/week, 1 serving/day, and ≥2 servings/day. Moreover, each question had a clear definition of serving size of the food item consumed [28].

### 2.7. Statistical Analysis

Dietary patterns were identified by factor analysis. Both the Kaiser–Meyer–Olkin index (0.827) and Bartlett’s test (*p* < 0.001) indicated that the correlation among the variables was sufficient for factor analysis. Orthogonal rotation with the Kaiser criterion (eigenvalues > 1.3) was used to determine the number of factors (also known as dietary patterns) [33]. Food items were retained in the pattern if the factor loading value was ≥0.30. Moreover, if the food item had a factor loading of ≥0.30 in different dietary patterns, the dietary pattern was determined by the one which had a higher factor loading value. The three factors derived (also known as dietary patterns) by principal component analysis (PCA) were named on the basis of the data interpretation. Factor scores were calculated for each dietary pattern by summing up the consumption of food items weighed by factor loadings. Within each dietary pattern, subjects were further divided into the tertile groups. Categorical variables are presented as a number or percentage, while continuous variables are presented as mean ± standard deviation (SD). For categorical data (gender, education, marital status, smoking, drinking, and physical activity), chi-squared test was used to compare the differences in the characteristics of the subjects among tertiles of dietary patterns. For continuous variables (age, BMI, TG, TC, LDL-C, HDL-C, and FPG), one-way analysis of variance (ANOVA) and Bonferroni post-hoc test were used for comparison. To evaluate the associations between dietary patterns across tertiles and dyslipidemia components or FPG, the multivariable logistic regression analysis was performed, and the odds ratios (ORs) and 95% confidence intervals (CIs) were calculated. In the regression model, we categorized the components of dyslipidemia and abnormal FPG as a high level of TG (≥1.7 mmol/L), a high level of TC (≥5.2 mmol/L), a high level of LDL-C (≥3.4 mmol/L), a low level of HDL-C (<1.04 mmol/L), and a high level of FPG (≥7.0 mmol/L). Two models were presented: model 1 represented crude data, while model 2 represented data after adjusting for gender, age, education, marital status, smoking, drinking, physical activity, and BMI. For the tertiles of each dietary pattern, the first tertile (T1) was considered as the reference group. Statistical analyses were conducted using SPSS 23 (IBM Corp., Armonk, NY, USA). All *p*-values presented are 2-tailed, and *p* < 0.05 was considered significant.

## 3. Results

Among 13,609 subjects with dyslipidemia and elevated FPG, 49.9% had elevated TG, 24.3% had elevated TC, 54.6% had elevated LDL-C, 24.4% had low HDL-C, 95.7% were in prediabetic status (5.6 mmol/L ≤ FPG < 7.0 mmol/L), and 4.3% were diabetic (FPG ≥ 7.0 mmol/L) (data not shown).

### 3.1. Dietary Patterns

Three dietary patterns were identified by PCA method as shown in Table 1. The meat–convenience dietary pattern (pattern I) explained 18.95% of the variance and consisted of nine food items: deep-fried food, processed food (i.e., ham/salami/sausage/canned products), sauces (i.e., soy sauce/spicy sauce/vinegar/ketchup), sugar-added beverages, meat (i.e., pork/beef/chicken/lamb/veal), instant noodles, organ meats (i.e., heart/liver/kidneys), rice or flour cooked in oil (i.e., fried rice or noodles), and eggs (i.e., chicken eggs, duck eggs, and quail eggs). The vegetables–fruits–seafood dietary pattern (pattern II) explained 10.51% of the variance and consisted of seven food items: dark- or light-colored vegetables (i.e., cauliflower/squash/cabbage/Chinese cabbage/carrot/cucumber/Chinese broccoli/spinach/broccoli), vegetables with oil or dressing, fruits, seafood (i.e., fish/shrimp/shellfish), legumes or soy products (i.e., soybean milk/tofu/beans), and rice or flour products (i.e., rice/noodles/pasta/plain bread/steamed buns). The dairy–complex carbohydrate (carb) dietary pattern (pattern III) explained 6.64% of the variance and consisted of six food items: dairy products (i.e., yogurt/cheese/fermented drinks), milk (i.e., liquid or powdered milk), refined dessert, root crops (i.e., sweet potatoes/potatoes/taro/corn/Chinese yam), jam or honey, and whole grains (i.e., brown rice, mixed grains, whole wheat bread, and oats).

### 3.2. Characteristics of the Subjects and Dietary Patterns

The characteristics of subjects across tertiles of dietary patterns are summarized in Table 2. Subjects from the higher tertiles of the meat–convenience dietary pattern were likely to be younger and heavier; had a greater proportion of individuals who were male, had higher education level, single, current smokers, drinkers, and physically inactive; and had higher TG level but lower TC, LDL-C, HDL-C, and FPG levels compared with those from the lowest tertile of the meat–convenience dietary pattern, while subjects from the higher tertiles of the vegetables–fruits–seafood dietary pattern were likely to be older and heavier; had a greater proportion of individuals who were male, had higher education level, married, nonsmokers, drinkers, and physically active; and had higher TG and FPG levels but lower TC level. By contrast, subjects from the higher tertiles of the dairy–complex carb dietary pattern were likely to be older and thinner; had a greater proportion of individuals who were female, had higher education level, married, nonsmokers, nondrinkers, and physically active; and had higher TC, LDL-C, and HDL-C levels, but lower TG and FPG levels.

### 3.3. Dietary Patterns, Dyslipidemia, and Abnormal Fasting Plasma Glucose

The odds ratios of components of dyslipidemia and abnormal FPG across tertiles of dietary patterns are shown in Table 3. Before adjustment (model 1), the highest tertile (T3) of the meat–convenience dietary pattern was significantly associated with increased risk of high TG level (OR: 1.436, 95% CI: 1.322–1.559, *p*-trend = 0.000) and low HDL-C level (OR: 1.498, 95% CI: 1.360–1.651, *p*-trend = 0.000), while being significantly associated with decreased risk of high TC level (OR: 0.777, 95% CI: 0.705–0.856, *p*-trend = 0.000) and high FPG level (OR: 0.815, 95% CI: 0.667-0.997, *p*-trend = 0.044) compared with the lowest tertile (T1). After adjusting for gender, age, education, marital status, smoking, drinking, physical activity, and BMI (model 2), the meat–convenience dietary pattern was not significantly correlated with components of dyslipidemia and FPG.

The highest tertile of the vegetables–fruits–seafood dietary pattern was significantly associated with increased risk of high TG level (OR: 1.131, 95% CI: 1.042–1.229, *p*-trend = 0.003) in the unadjusted model and high FPG level in both the unadjusted (OR: 1.358, 95% CI: 1.112–1.657, *p*-trend = 0.002) and adjusted models (OR: 1.324, 95% CI: 1.079–1.625, *p*-trend = 0.005), but was significantly associated with decreased risk of high TC level in both the unadjusted (OR: 0.883, 95% CI: 0.802–0.972, *p*-trend = 0.011) and adjusted models (OR: 0.893, 95% CI: 0.810–0.985, *p*-trend = 0.023) compared with the lowest tertile. After adjustment, LDL-C level was negatively correlated with the vegetables–fruits–seafood dietary pattern (*p*-trend = 0.049), but other dyslipidemic components such as a high level of TG and a low level of HDL-C were not significantly correlated with the vegetables–fruits–seafood dietary pattern.

The highest tertile of the dairy–complex carb dietary pattern was significantly associated with increased risk of high TC level (OR: 1.195, 95% CI: 1.086–1.315, *p*-trend = 0.000) compared with T1 in the unadjusted model, but was significantly associated with decreased risk of low HDL-C (OR: 0.808, 95% CI: 0.734–0.889, *p*-trend = 0.000) and high FPG levels (OR: 0.700, 95% CI: 0.570–0.860, *p*-trend = 0.001) in the unadjusted model. However, high TC and high FPG levels were not significantly correlated with the dairy–complex carb dietary pattern after adjustment, while the highest tertile of the dairy–complex carb dietary pattern was significantly associated with increased risk of high LDL-C level compared with T1 in both the unadjusted (OR: 1.239, 95% CI: 1.140–1.346, *p*-trend = 0.000) and adjusted models (OR: 1.171, 95% CI: 1.075–1.275, *p*-trend = 0.000), but was significantly associated with decreased risk of high TG level in both the unadjusted (OR: 0.665, 95% CI: 0.612-0.722, *p*-trend = 0.000) and adjusted models (OR: 0.815, 95% CI: 0.744–0.891, *p*-trend = 0.000).

## 4. Discussion

In this large cross-sectional study, specific Taiwanese dietary patterns were found to be associated with components of dyslipidemia and FPG level in young and middle-aged Taiwanese adults with dyslipidemia and elevated FPG. By using the factor analysis, three dietary patterns were derived: meat–convenience, vegetables–fruits–seafood, and dairy–complex carb dietary patterns. The meat–convenience dietary pattern was characterized by high consumption of deep-fried and processed food, sauces, sugar-added beverages, meat and organ meats, instant noodles, rice or flour cooked in oil, and eggs. This dietary pattern was quite similar to the Westernized or unhealthy dietary pattern as described in other studies [28,33]. In the unadjusted model, we found that the meat–convenience dietary pattern was inversely correlated with high TC and high FPG levels, but positively correlated with high TG and low HDL-C levels. These findings were consistent with those observed in a Western or unhealthy diet high in saturated fatty acids. High intake of saturated fatty acids has been shown to have a positive association with increased blood lipids and blood pressure [38]. Therefore, the meat–convenience dietary pattern is thought to be strongly correlated with the development of CVD [39]. However, these correlations became statistically nonsignificant after adjustment for gender, age, education, marital status, smoking, drinking, physical activity, and BMI in our study, indicating these confounding factors might reduce or cancel these associations as mentioned previously.

The vegetables–fruits–seafood dietary pattern, characterized by high consumption of vegetables, vegetables with oil or dressing, fruits, seafood, legumes, soy products, and rice or flour products, appeared to be inversely correlated with elevated TC and LDL-C levels, but positively correlated with abnormal FPG level. This dietary pattern is comparable to the heart-healthy Mediterranean dietary pattern, which emphasizes higher consumption of olive oil, vegetables, legumes, whole grains, fruits, and nuts, moderate consumption of fish and poultry, and low consumption of red meat and full-fat dairy products. The Mediterranean diet is a plant-based dietary pattern which has long been praised for its various health benefits, mainly related to decreased risk of CVD and cancer, as well as decreased all-cause and disease-specific mortality [30]. Moreover, recent randomized controlled trials also showed the Mediterranean diet as effective in reducing both systolic and diastolic blood pressure, TG, and LDL-C levels, and increasing HDL-C level [40]. Our results were consistent with previous studies done on the Mediterranean diet, where the vegetables–fruits–seafood dietary pattern was significantly associated with reduced TC and LDL-C levels and appeared to have some protective effects against dyslipidemia and CVD [40,41]. However, our results indicated that the vegetables–fruits–seafood dietary pattern was correlated with higher risk of abnormal FPG, which was different from other previous studies where the Mediterranean diet seemed to play a beneficial role in glucose metabolism [29,42]. The plausible explanation is that we did not assess the consumption of individual food items in these subjects, and these subjects may actually consume more simple carbohydrate food items such as fruits and rice/noodles/bread in their diet, which affected glucose homeostasis.

The dairy–complex carb dietary pattern, characterized by high consumption of dairy products, milk, refined dessert, root crops, jam or honey, and whole grains, was inversely correlated with elevated TG and low HDL-C levels, but positively correlated with elevated LDL-C level. The diagnosing criteria for both metabolic syndrome and dyslipidemia have two in common: elevated TG and low HDL-C concentrations. There are some plausible mechanisms explaining the beneficial effects of dairy components on metabolic syndrome variables. Dairy foods such as milk, cheese, and yogurt are rich in calcium, which may exert some positive effects on serum lipid profiles and obesity. Calcium intake at the dose of 2500 mg/day from low-fat dairy foods significantly increased fecal fat excretion and decreased mRNA expression of fatty acid synthase in the adipose tissue of healthy subjects [43]. Consistent with our findings, a cross-sectional study with 130,420 Korean adults enrolled demonstrated that higher consumption of milk (≥2 servings/day for females and ≥1 servings/day for males) was associated with reduced odds of metabolic syndrome components such as hypertriglyceridemia (OR: 0.76, 95% CI: 0.69–0.85 in females; OR: 0.91, 95% CI:0.84–0.99 in males) and lower HDL-C (OR: 0.61, 95% CI: 0.56–0.67 in females; OR: 0.84, 95% CI: 0.79–0.89 in males) [44]. Besides calcium, other nutrients found in dairy products, such as milk proteins and fatty acids, were claimed to be protective factors of metabolic syndrome in some observational studies [45,46,47]. Two major milk proteins in dairy foods are casein and whey protein, which may improve serum lipid profile by reducing the postprandial triglycerides [45]. Although dairy products are high in saturated fatty acids, consumption of dairy products was inversely associated with components of metabolic syndrome in both cross-sectional and prospective studies [46,47]. Besides abundant saturated fatty acids in dairy products, approximately 25% of milk fat is comprised by monounsaturated fatty acids such as oleic acid [48]. A monounsaturated fat-rich diet had beneficial results on the ratio of HDL-C:TC and tended to increase HDL-C level and reduce TG level [48]. Short-term human intervention studies [46,47] also demonstrated that subjects who consumed a high-fat diet enriched with dairy minerals such as calcium, potassium, and magnesium had significantly lower TC and LDL-C levels than those who consumed a control diet. Collectively, current evidence suggests that dairy consumption is correlated with improving serum lipid profiles and reduced risk of CVD, potentially mediated by fecal fat excretion [48].

In terms of glucose homeostasis, higher intake of dairy foods and dietary fiber were associated with better glycemic control shown by previous studies [48,49]. In addition, a dose–response meta-analysis of a prospective cohort study suggested an inverse correlation of dairy foods, particularly yogurt (80 g/day), with type 2 diabetes [49]. Yogurt may exert beneficial metabolic effects because of containing probiotics, which have been reported to lower blood cholesterol [50]. However, our study found no association between FPG level and the dairy–complex carb dietary pattern in the adjusted model. Since the inverse association between dairy food and type 2 diabetes was mostly observed due to higher yogurt and low-fat milk intake rather than high-fat milk or other dairy products such as cheese in other studies [49,51], our study could not establish a similar inverse correlation because our dairy food consumption category referred to all kinds of dairy foods instead (i.e., milk, cheese, yogurt, and other fermented dairy products).

Our study had several strengths. First, our study comprised of a large and ethnically homogeneous group of participants. Second, our study used dietary patterns determined by a posteriori method (i.e., factor analysis), which reflected true dietary habits better than the patterns determined on the basis of previously adopted dietary assumptions. Third, to the best of our knowledge, this was the first study in Taiwan to investigate the relationship between dietary patterns and components of dyslipidemia and abnormal FPG among a unique population—the young and middle-aged adult population (aged 20 to 50 years)—using a large sample size. However, the present investigation was not without weaknesses. First, it was limited by its cross-sectional design, which hindered any causal relationship. More observational and clinical intervention studies are warranted to examine our findings and develop specific dietary strategies for prevention and management of dyslipidemia and abnormal FPG. Second, the self-reported FFQ used at the MJ screening center only provided estimated information of habitual food intake rather than the actual consumption of nutrients and total caloric intake. Future studies should consider revising the structure of the self-reported FFQ to include a wider range of food items and to identify the composition of nutrients and the type of fats consumed. Third, the three dietary patterns derived from factor analysis explained only 36.1% of the total variation, which may not thoroughly address all Taiwanese dietary patterns. Furthermore, the use of a self-administered questionnaire might pose some potential self-reporting bias (i.e., underestimating portion sizes), which could be improved by using a well-trained interviewer to collect data from the subjects for increasing accuracy and consistency of the results. Fourth, the unavailability of glycated hemoglobin (HbA1C) data is part of the study limitation. Comparing to fasting blood glucose, HbA1C is an important indicator of an individual’s overall glycemic control over the past three months and has a strong correlation to the development of long-term diabetes complications [52]. Lastly, we suggest that future studies including randomized clinical trials are needed to confirm the association between dietary pattern and components of dyslipidemia and fasting plasma glucose.

## 5. Conclusions

Our findings support that the dietary pattern may have a role in the prevention and management of dyslipidemia and abnormal fasting plasma glucose. The vegetables–fruits–seafood dietary pattern is inversely associated with hypercholesterolemia and positively associated with hyperglycemia. The dairy–complex carb dietary pattern is inversely correlated with hypertriglycemia and low HDL-C level.

## Figures and Tables

**Table 1:** Factor loadings and dietary patterns derived by principal component analysis for the 22 food groups ^1^.

Food Groups	Factor I Meat–Convenience Dietary Pattern	Factor II Vegetables–Fruits–Seafood Dietary Pattern	Factor III Dairy–Complex Carb Dietary Pattern
Milk	-	-	0.662
Dairy products	-	-	0.692
Eggs	0.412	-	-
Meat	0.536	-	-
Organ meats	0.475	-	-
Legumes/soy products	-	0.370	-
Seafood	-	0.400	-
Light-colored vegetables	-	0.800	-
Dark-colored vegetables	-	0.812	-
Fruits	-	0.423	0.373
Vegetables with oil/dressing	-	0.650	-
Rice/flour products	-	0.350	-
Whole grains	-	-	0.315
Root crops	-	0.338	0.443
Refined dessert	0.309	-	0.540
Jam/honey	0.348	-	0.412
Sugar-added beverages	0.536	-	-
Rice/flour cooked in oil	0.462	-	-
Deep-fried food	0.706	-	-
Instant noodles	0.512	-	-
Processed food	0.645	-	-
Sauces	0.618	-	-

^1^ Factor loading values <0.30 were excluded for simplicity. Carb: carbohydrate.

**Table 2:** Characteristics of study participants across tertiles of dietary patterns (*n* = 13,609)^1^.

Variables	Meat–Convenience Dietary Pattern				Vegetables–Fruits–Seafood Dietary Pattern				Dairy–Complex Carb Dietary Pattern			
	T1 (*n* = 4536)	T2 (*n* = 4537)	T3 (*n* = 4536)	*p^2^*	T1 (*n* = 4536)	T2 (*n* = 4537)	T3 (*n* = 4536)	*p^2^*	T1 (*n* = 4536)	T2 (*n* = 4537)	T3 (*n* = 4536)	*p^2^*
Factor score ranges of dietary patterns	−3.670 to −0.452	−0.452 to 0.348	0.348 to 5.305		−3.647 to −0.397	−0.397 to 0.246	0.246 to 4.651		−3.296 to −0.520	−0.520 to 0.330	0.330 to 5.441	
Gender				0.000				0.000				0.000
Male (%)	2941 (64.8)	3531 (77.8)	3833 (84.5)		3285 (72.4)	3524 (77.7)	3496 (77.1)		3579 (78.9)	3405 (75.0)	3321 (73.2)	
Female (%)	1595 (35.2)	1006 (22.2)	703 (15.5)		1251 (27.6)	1013 (22.3)	1040 (22.9)		957 (21.1)	1132 (25.0)	1215 (26.8)	
Age (years)	41.3 ± 6.3	39.2 ± 6.5	37.0 ± 6.6	0.000	38.7 ± 6.9	39.3 ± 6.6	39.4 ± 6.6	0.000	38.7 ± 6.9	39.1 ± 6.6	39.6 ± 6.5	0.000
Education				0.000				0.000				0.000
Below university level	2909 (64.1)	2689 (59.3)	2697 (59.5)		2904 (64.0)	2731 (60.2)	2660 (58.6)		3085 (68.0)	2738 (60.3)	2472 (54.5)	
University level or above	1627 (35.9)	1848 (40.7)	1839 (40.5)		1632 (36.0)	1806 (39.8)	1876 (41.4)		1451 (32.0)	1799 (39.7)	2064 (45.5)	
Marital status				0.000				0.000				0.000
Single	804 (17.7)	847 (18.7)	1182 (26.1)		1090 (24.0)	884 (19.5)	859 (18.9)		1058 (23.3)	894 (19.7)	881 (19.4)	
Married	3549 (78.2)	3554 (78.3)	3228 (71.2)		3271 (72.1)	3522 (77.6)	3538 (78.0)		3310 (73.0)	3508 (77.3)	3513 (77.4)	
Divorced/widowed	183 (4.0)	136 (3.0)	126 (2.8)		175 (3.9)	131 (2.9)	139 (3.1)		168 (3.7)	135 (3.0)	142 (3.1)	
Smoking				0.000				0.003				0.000
No	3670 (80.9)	3244 (71.5)	2696 (59.4)		3122 (68.8)	3221 (71.0)	3267 (72.0)		2830 (62.4)	3271 (72.1)	3509 (77.4)	
Yes	866 (19.1)	1293 (28.5)	1840 (40.6)		1414 (31.2)	1316 (29.0)	1269 (28.0)		1706 (37.6)	1266 (27.9)	1027 (22.6)	
Drinking				0.000				0.008				0.000
<2 times/week	3780 (83.3)	3527 (77.7)	3249 (71.6)		3585 (79.0)	3508 (77.3)	3463 (76.3)		3255 (71.8)	3574 (78.8)	3727 (82.2)	
≥2 times/week	756 (16.7)	1010 (22.3)	1287 (28.4)		951 (21.0)	1029 (22.7)	1073 (23.7)		1281 (28.2)	963 (21.2)	809 (17.8)	
Physical activity				0.000				0.000				0.000
<150 min/week	3613 (79.7)	3733 (82.3)	3822 (84.3)		3907 (86.1)	3686 (81.2)	3575 (78.8)		3936 (86.8)	3760 (82.9)	3472 (76.5)	
≥150 mins/week	923 (20.3)	804 (17.7)	714 (15.7)		629 (13.9)	851 (18.8)	961 (21.2)		600 (13.2)	777 (17.1)	1064 (23.5)	
BMI (kg/m^2^)	24.3 ± 3.3	24.8 ± 3.4	25.2 ± 3.6	0.000	24.6 ± 3.5	24.7 ± 3.4	24.9 ± 3.5	0.000	25.1 ± 3.6	24.7 ± 3.5	24.4 ± 3.3	0.000
TG (mmol/L)	1.7 ± 0.8	1.8 ± 0.8	1.9 ± 0.9	0.000	1.7 ± 0.9	1.8 ± 0.9	1.8 ± 0.9	0.001	1.9 ± 0.9	1.8 ± 0.8	1.7 ± 0.8	0.000
TC (mmol/L)	5.6 ± 0.8	5.5 ± 0.8	5.6 ± 0.8	0.015	5.6 ± 0.8	5.6 ± 0.8	5.5 ± 0.8	0.001	5.5 ± 0.8	5.6 ± 0.8	5.6 ± 0.8	0.014
LDL-C (mmol/L)	3.3 ± 1.0	3.2 ± 1.1	3.2 ± 1.1	0.000	3.3 ± 1.1	3.3 ± 1.1	3.3 ± 1.0	0.634	3.2 ± 1.1	3.3 ± 1.1	3.3 ± 1.0	0.000
HDL-C (mmol/L)	1.33 ± 0.47	1.25 ± 0.46	1.21 ± 0.45	0.000	1.27 ± 0.48	1.25 ± 0.46	1.27 ± 0.45	0.558	1.24 ± 0.45	1.26 ± 0.47	1.29 ± 0.47	0.000
FPG (mmol/L)	6.03 ± 1.06	5.98 ± 0.89	5.99 ± 0.95	0.043	5.99 ± 0.93	5.99 ± 0.89	6.03 ± 1.07	0.040	6.04 ± 1.03	6.00 ± 0.96	5.96 ± 0.90	0.000

BMI: body mass index, TG: triglycerides, TC: total cholesterol, LDL-C: low-density lipoprotein cholesterol, HDL-C: high-density lipoprotein cholesterol, FPG: fasting plasma glucose. ^1^ Data are presented as the mean ± SD for continuous variables and *n* (%) for categorical variables. ^2^ The *p*-values were derived from general linear regression for continuous variables and from chi-squared test for categorical variables.

**Table 3:** Odds ratios (95% confidence intervals) for components of dyslipidemia^1^ and abnormal fasting plasma glucose^2^ across tertiles of dietary patterns.

		Meat–Convenience Dietary Pattern		Vegetables–Fruits–Seafood Dietary Pattern		Dairy–Complex Carb Dietary Pattern	
Variables	Tertiles	Model 1 ^3^	Model 2 ^4^	Model 1	Model 2	Model 1	Model 2
High level of TG	T1	1	1	1	1	1	1
	T2	1.161 (1.069–1.260)	0.955 (0.873–1.045)	1.076 (0.991–1.169)	1.040 (0.952–1.136)	0.794 (0.731–0.862)	0.907 (0.830–0.991)
	T3	1.436 (1.322–1.559)	1.024 (0.932–1.125)	1.131 (1.042–1.229)	1.082 (0.990–1.182)	0.665 (0.612–0.722)	0.815 (0.744–0.891)
	*p-*Trend	0.000	0.616	0.003	0.081	0.000	0.000
High level of TC	T1	1	1	1	1	1	1
	T2	0.847 (0.768–0.933)	0.968 (0.875–1.070)	0.950 (0.863–1.047)	0.959 (0.869–1.058)	1.145 (1.041–1.259)	1.066 (0.968–1.175)
	T3	0.777 (0.705–0.856)	0.997 (0.899–1.107)	0.883 (0.802–0.972)	0.893 (0.810–0.985)	1.195 (1.086–1.315)	1.063 (0.962–1.174)
	*p*	0.000	0.979	0.011	0.023	0.000	0.224
High level of LDL-C	T1	1	1	1	1	1	1
	T2	0.995 (0.916–1.081)	1.030 (0.946–1.121)	0.996 (0.917–1.082)	0.969 (0.892–1.054)	1.185 (1.091–1.287)	1.147 (1.055–1.248)
	T3	0.981 (0.904–1.066)	1.082 (0.990–1.182)	0.945 (0.870–1.026)	0.920 (0.846–1.000)	1.239 (1.140–1.346)	1.171 (1.075–1.275)
	*p-*Trend	0.658	0.084	0.177	0.049	0.000	0.000
Low level of HDL-C	T1	1	1	1	1	1	1
	T2	1.235 (1.119–1.363)	1.028 (0.927–1.140)	1.046 (0.951–1.151)	1.031 (0.934–1.139)	0.876 (0.797–0.963)	0.939 (0.850–1.036)
	T3	1.498 (1.360–1.651)	1.076 (0.968–1.196)	0.978 (0.889–1.077)	0.955 (0.863–1.055)	0.808 (0.734–0.889)	0.905 (0.818–1.002)
	*p-*Trend	0.000	0.172	0.660	0.359	0.000	0.048
High level of FPG	T1	1	1	1	1	1	1
	T2	0.825 (0.675–1.008)	0.898 (0.729–1.105)	0.959 (0.774–1.188)	0.940 (0.755–1.170)	0.844 (0.694–1.027)	0.955 (0.780–1.169)
	T3	0.815 (0.667–0.997)	0.934 (0.750–1.162)	1.358 (1.112–1.657)	1.324 (1.079–1.625)	0.700 (0.570–0.860)	0.832 (0.671–1.031)
	*p-*Trend	0.044	0.517	0.002	0.005	0.001	0.098

TG: triglycerides, TC: total cholesterol, LDL-C: low-density lipoprotein cholesterol, HDL-C: high-density lipoprotein cholesterol, FPG: fasting plasma glucose. The odds ratios across tertiles of dietary patterns were compared with that in the reference group (T1). ^1^ Components of dyslipidemia were defined as a high level of TG (≥1.7 mmol/L), a high level of TC (≥5.2 mmol/L), a high level of LDL-C (≥3.4 mmol/L), or a low level of HDL-C (<1.04 mmol/L). ^2^ Abnormal fasting plasma glucose was defined as ≥7.0 mmol/L. ^3^ Model 1: unadjusted model. ^4^ Model 2: adjusted for gender, age, education, marital status, smoking, drinking, physical activity, and BMI.
